# A harmonized analysis of five Canadian pregnancy cohort studies: exploring the characteristics and pregnancy outcomes associated with prenatal alcohol exposure

**DOI:** 10.1186/s12884-023-05447-2

**Published:** 2023-02-28

**Authors:** Rose A. Schmidt, Tina W. Wey, Kelly D. Harding, Isabel Fortier, Stephanie Atkinson, Suzanne Tough, Nicole Letourneau, Julia A. Knight, William D. Fraser, Alan Bocking

**Affiliations:** 1 Dalla Lana School of Public Health, University of Toronto, Toronto, ON Canada; 2 Institute for Mental Health Policy Research, Centre for Addiction and Mental Health (CAMH), Toronto, ON Canada; 3 Research Institute of the McGill University Health Centre, Montreal, QC Canada; 4 Canada Fetal Alcohol Spectrum Disorder Research Network, Vancouver, BC Canada; 5 Department of Psychology, Laurentian University, Sudbury, ON Canada; 6 Department of Pediatrics, McMaster University, Hamilton, ON Canada; 7 Owerko Centre at the Alberta Children’s Hospital Research Institute, University of Calgary, Calgary, AB Canada; 8 Cumming School of Medecine, University of Calgary, Calgary, AB Canada; 9 Lunenfeld-Tanenbaum Research Institute, Sinai Health, Toronto, ON Canada; 10 Department of Obstetrics and Gynecology, University of Sherbrooke, Sherbrooke, QC Canada; 11 Department of Obstetrics and Gynecology, University of Toronto, Toronto, ON Canada

**Keywords:** Prenatal alcohol exposure, Data harmonization, Alcohol, Pregnancy, Birth outcomes

## Abstract

**Background:**

As a teratogen, alcohol exposure during pregnancy can impact fetal development and result in adverse birth outcomes. Despite the clinical and social importance of prenatal alcohol use, limited routinely collected information or epidemiological data exists in Canada. The aim of this study was to pool data from multiple Canadian cohort studies to identify sociodemographic characteristics before and during pregnancy that were associated with alcohol consumption during pregnancy and to assess the impact of different patterns of alcohol use on birth outcomes.

**Methods:**

We harmonized information collected (e.g., pregnant women’s alcohol intake, infants' gestational age and birth weight) from five Canadian pregnancy cohort studies to consolidate a large sample (*n* = 11,448). Risk factors for any alcohol use during pregnancy, including any alcohol use prior to pregnancy recognition, and binge drinking, were estimated using binomial regressions including fixed effects of pregnancy cohort membership and multiple maternal risk factors. Impacts of alcohol use during pregnancy on birth outcomes (preterm birth and low birth weight for gestational) were also estimated using binomial regression models.

**Results:**

In analyses adjusting for multiple risk factors, women’s alcohol use during pregnancy, both any use and any binge drinking, was associated with drinking prior to pregnancy, smoking during pregnancy, and white ethnicity. Higher income level was associated with any drinking during pregnancy. Neither drinking during pregnancy nor binge drinking during pregnancy was significantly associated with preterm delivery or low birth weight for gestational age in our sample.

**Conclusions:**

Pooling data across pregnancy cohort studies allowed us to create a large sample of Canadian women and investigate the risk factors for alcohol consumption during pregnancy. We suggest that future pregnancy and birth cohorts should always include questions related to the frequency and amount of alcohol consumed before and during pregnancy that are prospectively harmonized to support data reusability and collaborative research.

**Supplementary Information:**

The online version contains supplementary material available at 10.1186/s12884-023-05447-2.

## Background

Alcohol is a widely used substance among women of reproductive age in Canada [1]. Alcohol use during pregnancy can cause miscarriage [2] and low birth weight [3], and, as a teratogen, can impact fetal development [4]. Prenatal alcohol exposure has been causally linked to detrimental outcomes on cognition [5], as well as contributing to the potential risk of low birthweight and preterm birth depending on the amount of alcohol consumed. Fetal alcohol spectrum disorder (FASD) is a diagnostic term that describes a range of physical and cognitive deficits resulting from alcohol use during pregnancy [6, 7]. Although larger amounts of alcohol cause greater harm [2, 8], even a small amount of alcohol during pregnancy has been linked to behavioural problems among children [9, 10].

Current best practice and alcohol use guidelines, such as those intended to optimize patient care in obstetrics and gynecology practice, recommend encouraging abstinence from alcohol during pregnancy [11–13]. Most women also report reducing alcohol intake during pregnancy due to concerns about fetal development [14]. However, evidence suggests that at least 10% of Canadian women^1^ consume alcohol during pregnancy [14, 15]. Further, many pregnancies may be exposed to alcohol prior to pregnancy identification. Women often continue their usual pattern of alcohol consumption into the early weeks of an unplanned pregnancy [16–18], and almost one in five Canadian women aged 18–34 report heavy drinking, or having 4 or more drinks, on one occasion, at least once a month in the past year [19].

In Canada, routinely collected information or epidemiological data regarding alcohol use during pregnancy is limited [20] and there is no consistent approach across the provinces or territories to collect and record this information on antenatal record forms [21]. Provincial databases and registries, such as BORN Ontario, sometimes capture information about alcohol use during pregnancy [22], which may be considered part of routine surveillance if information about alcohol use during pregnancy is collected and documented by prenatal care providers. Recent findings indicate that 95% of health care providers across Canada report asking women about their alcohol consumption, but only 45% report using a standardized screening tool to do so [23]. However, research exploring routine questioning indicates that implementation varies even within countries with national programming for screening and brief intervention (e.g., Scotland) [24], and reinforces the importance of aspects beyond forms, such as how the conversation is framed and constructed to facilitate self-report.

Surveillance questions are typically collected and documented using one of three approaches: framing questions about alcohol use during pregnancy using basic yes/no questions; capturing information numerically, such as asking about the number of drinks per day, before or during pregnancy, or per week; or using a full comprehensive checklist [21]. Not only does Canada not collect data via routine surveillance but reporting also relies predominantly on the national *Maternity Experience Survey* (MES) conducted in 2006 [25], as well as a small number of questions about alcohol use during pregnancy in the annual Canadian Community Health Survey, which does not specifically sample pregnant people, or inconsistent provincial perinatal health forms [26]. The results of the MES were published in 2008, yet data from that survey continue to drive the narrative regarding the prevalence of alcohol use during pregnancy in Canada, as well as the factors that influence alcohol use during pregnancy. Although previous systematic reviews have suggested that low to moderate levels of alcohol consumption do not have an impact on either birth weight or preterm birth rates, the results from individual studies are quite variable. We therefore chose to study the association of prenatal alcohol exposure with birth weight and preterm birth rates in this analysis [27, 28].

Pooling data from multiple studies can increase statistical power to detect associations and allows for answering novel research questions that may not have been the focus of the original studies [29, 30]. When the variable of interest has prevalence, as in the case of heavy alcohol use or binge drinking during pregnancy, pooling data from several studies may be necessary to attain a large enough sample size to fit a statistical model of interest [31]. However, combining data across studies requires careful data harmonization to make data items collected by different studies comparable [32]. This can be challenging in retrospective initiatives, where individual studies have different research objectives and apply different methods. In practice, data harmonization requires finding a balance between targeting precise concepts to address research objectives when minimal heterogeneity across studies exists and accepting greater heterogeneity to include more studies [33].

In response to the challenges related to pooling data across studies, Maelstrom Research proposed guidelines for rigorous data harmonization and documentation [30]. Drawing on this and other tools and expertise [32, 34, 35], the Research Advancement through Cohort Cataloguing and Harmonization (ReACH) initiative was established to provide a unique opportunity to leverage Canadian pregnancy cohort data regarding women’s alcohol use during pregnancy [36]. Multiple cohorts participating in ReACH collected information required for the current project regarding women's alcohol consumption patterns before pregnancy, prior to pregnancy recognition and during pregnancy.

The aims of this harmonized cohort study were to: 1) identify sociodemographic characteristics before and during pregnancy that were associated with alcohol consumption during pregnancy; and 2) assess the impact of different patterns of alcohol use on preterm birth and low birth weight for gestational age.

## Methods

### Characteristics of included cohort studies

When selected in 2018, there were eight cohorts in the ReACH Catalogue with required information regarding women’s alcohol use. To be included in this study, the cohorts had to meet the following inclusion criteria: 1) collect information on alcohol use before and during pregnancy; 2) collect birth weight and gestational age at delivery; 3) include at least 500 women; and 4) agree to participation in the project by sharing data. Of the eight cohorts, five met these criteria and were included in the current analysis. The cohorts participating in this study include the 3D Cohort Study (Design, Develop, Discover) [37], the All Our Families (AOF; formerly All Our Babies) pregnancy cohort [38], the Alberta Pregnancy Outcomes and Nutrition (APrON) cohort study [39], the Family Atherosclerosis Monitoring In earLY life (FAMILY) study [40], and the Ontario Birth Study (OBS) [41]. These cohorts are based in the provinces of Alberta, Ontario, and Quebec, and, at the time of the current study, included data collected from 2004–2019. The total number of women enrolled in these five studies at the time of analysis was 12,059. The number of women included in the harmonization process (i.e., with the information provided by cohorts for the current study) was *n* = 11,448 (3D *n* = 2,365, AOF *n* = 3,341, APrON *n* = 2,187, FAMILY *n* = 855 and OBS *n* = 2,700).

### Harmonization process

The Maelstrom guidelines for retrospective data harmonization were used to guide the harmonization process [30] following a series of iterative steps: 1) define the research questions, objectives, and protocol; 2) assemble pre-existing information and select studies; 3) define targeted variables and evaluate harmonization potential; 4) process data; 5) estimate quality of the harmonized dataset(s) generated; and 6) disseminate and preserve final harmonization products. The Prenatal Alcohol Exposure (PAE) project investigators (RS, KH, AB) developed the initial research objectives, protocol, and analysis plan that guided harmonization decisions. A central harmonization team (including data analysts, statisticians, and epidemiologists) at Maelstrom Research (Research Institute of the McGill University Health Centre) was responsible for implementing harmonization procedures and generating harmonized data and related documentation. All harmonization decisions were made in discussion with the PAE project investigators.

#### Harmonization procedures implemented

The harmonization team assembled documentation and information about potential participating studies, explored the variables collected by studies in detail, identified harmonization challenges, and drafted the list of variables required to address the research questions. This initial list of variables was discussed with the research team to produce the first version of the DataSchema (i.e., the list and definition of the variables to be generated across studies). The harmonization team then formally evaluated the ability for each study to generate each DataSchema variable (the harmonization potential) and identified the study-specific variables required to generate the DataSchema variables.

Access to study-specific variables was requested from each individual study following local institutional data access procedures and ethical and legal requirements. Ethics approval was also obtained from the Research Institute of the McGill University Health Center Research Ethics Board (#2019–5058). When approval was received, de-identified individual participant data (IPD) was transferred from each study-specific server through a secure network to an Opal server at PolicyWise for Children and Families (https://policywise.com/).

#### Harmonized variables generated

The harmonization team assessed the variables received from each study for completeness and to understand content. The harmonization potential for each DataSchema variable was verified based on data received and, where required, questions were discussed with study data managers.

For each DataSchema variable that was deemed possible to generate, R scripts were written to process the study-specific variables into the harmonized format [42, 43]. The algorithms, harmonization logic, and content of harmonized variables were validated, and the final harmonization status for each DataSchema variable for each study was documented as either complete or impossible. Variables in the ReACH Prenatal Alcohol Exposure Harmonization Project are documented on the Maelstrom website [44].

Table 1 provides an overview of key harmonized variables and their availability in each cohort. Exposure frequency and quantity of alcohol consumption at different time points during pregnancy were originally of interest, but heterogeneity across studies in timing of data collection events and questionnaire wording made it impossible to define harmonized variables of acceptable quality. Thus, the main exposure variable in our analysis was any alcohol consumption during pregnancy, which was defined as any instance of women’s alcohol intake during pregnancy (yes/no). Binge drinking during pregnancy was defined as women’s consumption of either “four or more” or “five or more” more drinks on one occasion at any point during pregnancy (yes/no), depending on the cohort. Despite “four or more” being a consistent definition with the sex-specific Canadian Low Risk Drinking Guidelines for special occasion drinking [45], most (3/5) of the cohorts used “five or more”.

**Table 1:** Variables where harmonization was possible, across cohorts

Harmonized Variable		AOF^a^	APrON^b^	FAMILY^c^	OBS^d^	3D^e^
**Maternal behaviors and risk factors**						
**Alcohol use**						
Alcohol consumption before pregnancy (yes/no)		-^f^	X^g^	-	X	-
Alcohol consumption during year prior to pregnancy (yes/no)		X	-	X	X	X
Alcohol consumption during pregnancy (yes/no)		X	X	X	X	X
Binge drinking during year prior to pregnancy (yes/no)^*h*^		X	-	-	X	X
Binge drinking during pregnancy (yes/no)		X	-	X	X	X
** Age**						
Age at first questionnaire response during current pregnancy (integer)		X	-	X	X	X
Age at delivery for current pregnancy/birth (integer)		X	X	X	-	X
** Ethnicity**						
Ethnic background – White (yes/no)		X	X	X	X	X
** Marital status**						
Marital status (categorical)		X	X	-	X	X
** Education**						
Highest level of education completed (categorical)		X	X	-	X	X
** Employment**						
Employment status (yes/no)		X	-	X	X	X
** Income**						
Income level (categorical)		X	X	X	X	X
**Smoking**						
Cigarette smoking before pregnancy (yes/no)		-	X	X	X	X
Cigarette smoking during pregnancy (yes/no)		X	X	X	X	X
** Physical Measures**						
BMI before pregnancy (continuous)		X	X	X	X	X
**Current Pregnancy**						
**Maternal reproductive history**						
Gravidity (integer)		X	X	X	X	X
Previous live births (integer)		X	X	X	X	X
**Maternal co-morbidities**						
Gestational hypertension (yes/no)		X	X	X	X	X
Gestational diabetes (yes/no)		X	X	X	X	X
Pre-eclampsia or eclampsia (yes/no)		X	X	X	X	-
**Birth outcomes**^**i**^						
Live birth (yes/no)		X	X	X	X	X
Gestational age at delivery (integer)		X	X	X	X	X
Birth weight (yes/no)		X	X	X	X	X

^a^All Our Families

^b^Alberta Pregnancy Outcomes and Nutrition

^c^Family Atherosclerosis Monitoring In earLY life

^d^Ontario Birth Study

^e^3D Study—Design, Develop, Discover

^f^**-**Variable not possible to harmonize

^g^ X Variable was harmonized

^h^ Binge drinking was defined as consuming 5 or more drinks on one occasion in AOF, APrON, FAMILY, and 3D, and as consuming 4 or more drinks on o ne occasion in OBS

^i^ Birth outcomes focus on singleton births or the first of a multiple birth

Other maternal alcohol use variables created included: consumption of alcohol at any point before pregnancy (yes/no), consumption of alcohol during the 12 months prior to pregnancy (yes/no), and binge drinking during the 12 months prior to pregnancy (yes/no). Variables about alcohol dependence or disorders were not possible to harmonize. Only two cohorts asked directly about alcohol use problems, but with questions targeting different aspects of problematic use. For example, AOF asked if participants had ever had alcohol dependency problems and ever sought treatment, and 3D asked if participants had ever felt annoyed by people criticizing their alcohol consumption or ever felt they should cut down.

Maternal risk factor variables included sociodemographic characteristics (e.g., maternal age, body mass index (BMI), ethnic background, marital status, education, income level), smoking behaviour, and reproductive history (for full specifications of variables, please refer to Supplementary Table S1). Birth outcome variables included gestational age at delivery and birth weight. Preterm birth was considered as delivery before 37 weeks’ gestation and small for gestational age was defined as infants at or below the 10^th^ percentile in birth weight compared to infants of the same sex and gestational age. To calculate low birth weight for gestational age, we used the reference ranges described by Kramer et al. 2001 [46].

### Statistical analysis

Pooled and study-specific harmonized variables were initially examined via descriptive statistics and bivariate analyses. Frequency of maternal alcohol use behaviours and maternal risk factors (e.g., sociodemographic characteristics, smoking behaviour, BMI, gravidity, and previous live births) were described, and unadjusted odds ratios were calculated for the relationship between maternal risk factors and alcohol use during pregnancy.

Risk factors for alcohol use during pregnancy (any alcohol consumption (1/0) and binge drinking (1/0)) were estimated using adjusted binomial regressions with maximum likelihood estimation. The models included fixed effects of cohort membership (dummy coded with OBS as the reference group) and multiple maternal risk factors. As not all risk factors of interest could be harmonized for all cohorts, a single model including all cohorts and risk factors of interest was not feasible.

We report results from one model that included complete cases (excluding observations with missing values) for four of five cohorts that included fixed effects of any alcohol use in the year prior to pregnancy (1/0), any smoking during pregnancy (1/0), ethnic background white (1/0), income level (categorical; reference Level 1 – Below Market Basket Measure (MBM), Level 2 – Above MBM but below median household income, Level 3 – Above median household income), and linear and quadratic terms for maternal age (years) [47]. A quadratic term for maternal age was included because initial exploration suggested a nonlinear association between age and alcohol use. Exploration of other model specifications with different combinations of risk factors and cohorts included indicated that direction and size of adjusted estimates were similar across models. Only variables available in at least three cohorts were used in pooled analyses. Potential heterogeneity of effects across cohorts was explored by fitting a model with all two-way interactions between cohort membership and all risk factors.

Analysis of birth outcomes was restricted to singleton births. Impacts of alcohol use during pregnancy on the birth outcomes of preterm birth (1/0) and low birth weight for gestational age (1/0) were then estimated using adjusted binomial regression models including fixed effects of either any alcohol consumption (1/0) or binge drinking (1/0) (in separate models), cohort membership, and the same set of maternal risk factors specified above. We considered $\propto =$ 0.05 statistically significant, but also report and make inferences based on 95% confidence intervals (CI). All analyses were conducted with R statistical software [42] in the RStudio computing environment.

## Results

### Descriptive statistics

A total of *n* = 11,399 women from the five cohorts were included in the harmonized dataset for analysis. Table 2 presents the frequencies and percentages of alcohol-related variables for each cohort. Although measures of frequency and amount of alcohol consumed before and during pregnancy, as well as an indication of the trimester in which alcohol was consumed, were part of the initial harmonization and analysis proposals, these harmonized variables could not be created due to limitations of available data. Instead, only dichotomous variables of any alcohol consumption or binge drinking before and during pregnancy could be created (see Table 1, Supplementary Table S1).

**Table 2:** Frequencies and percentages of alcohol use variable values across cohorts

	**AOF**			**APrON**				**FAMILY**				**OBS**				**3D**		
	(*N* = 3,341)^a^			(*N* = 2,187)				(*N* = 806)				(*N* = 2,700)				(*N* = 2,365)		
	**n**	**% total**^**b**^	**% observed**^c^	**n**	**% total**	**% observed**	**n**		**% total**	**% observed**	**n**		**% total**	**% observed**	**n**		**% total**	**% observed**
**Prior to Pregnancy**																		
**Ever drank alcohol**																		
No				261	11.9%	12.4%					203		7.5%	7.9%				
Yes				1836	84.0%	87.6%					2382		88.2%	92.1%				
Missing				90	4.1%	-					115		4.3%	-				
**Ever drank (year prior)**																		
No	590	17.7%	17.8%				267		33.1%	33.5%	122^*e*^		4.5%	5.1%	461		19.5%	19.5%
Yes	2728	81.7%	82.2%				530		65.8%	66.5%	2247		83.2%	94.9%	1898		80.3%	80.5%
Missing	23	0.7%	-				9		1.1%	-	331		12.3%	-	6		0.3%	-
**Binge drank (year prior)**^***d***^																		
No	1300	38.9%	49.1%								941		34.9%	40.4%	927		39.2%	48.9%
Yes	1349	40.4%	50.9%								1390		51.5%	59.6%	967		40.9%	51.1%
Missing	692	20.7%	-								369		13.7%	-	471		19.9%	-
**During Pregnancy**																		
**Ever drank alcohol**																		
No	1489	44.6%	51.0%	1952	89.3%	92.5%	768		95.3%	96.4%	1555		57.6%	75.0%	1050		44.4%	47.1%
Yes	1429	42.8%	49.0%	157	7.2%	7.4%	29		3.6%	3.6%	518		19.2%	25.0%	1180		49.9%	52.9%
Missing	423	12.7%	-	78	3.7%	-	9		1.1%	-	627		23.2%	-	135		5.7%	-
**Ever binge drank**																		
No	2593	77.6%	89.5%				797		98.9%	100.0%	2303		85.3%	99.2%	1385		58.6%	88.3%
Yes	304	9.1%	10.5%				0		0.0%	0.0%	18		0.7%	0.8%	183		7.7%	11.7%
Missing	444	13.3%	-				9		1.1%	-	379		14.0%	-	797		33.7%	-

^a^N = Number of participants from each cohort included in the harmonized dataset

^b^% total: percent of all participants

^c^% observed: percent of participants with non-missing values

^d^Binge drinking was defined as consuming 5 or more drinks on one occasion in AOF, APrON, FAMILY, and 3D, and as consuming 4 or more drinks on one occasion in OBS

^e^Frequencies for Ever drank in the year prior to pregnancy in OBS were taken from the variable as collected by the study (rather than creating a derived variable incorporating information from the separate variable about Ever drank alcohol prior to pregnancy) to be as compatible as possible with other studies. Hence the number of No responses appears lower than for Ever drank alcohol in this cohort

One cohort did not include data on women’s alcohol consumption in the one year prior to pregnancy, but only included a measure of any past alcohol consumption. Two cohorts did not measure binge drinking prior to pregnancy. One of these cohorts also did not include a measure of binge drinking during pregnancy, and in the other, 100% of the women who responded reported that they did not binge drink during pregnancy. Study differences in data collection and question wording likely affected the frequency of women reporting drinking at any time during pregnancy (Supplementary Table S2).

Table 3 describes the maternal characteristics of the subset of women who had data available for the variable “Ever drank during pregnancy” and were thus considered in unadjusted analyses (*n* = 10,127 women). Most of the participants were white (overall 76.7%), married or living with a partner (overall 95.6%), and highly educated (overall 97.9% completed high school, 86.3% completed post-secondary education). The overall median age for the combined sample was 32 years old (IQR = 6). About one third of the participants were enrolled in the cohort for their first pregnancy (overall 38.6%).

**Table 3:** Maternal characteristics of participants with non-missing values for the variable ‘ever drank alcohol during pregnancy’

	**AOF**		**APrON**		**FAMILY**		**OBS**		**3D**		**Total**	
	(*N* = 2,918)^a^		(*N* = 2,109)		(*N* = 797)		(*N* = 2,073)		(*N* = 2,230)		(*N* = 10,127)	
	**n**	**%**	**n**	**%**	**n**	**%**	**n**	**%**	**n**	**%**	**n**	**%**
Ever drank alcohol during pregnancy	1429	49.0	157	7.6	29	3.6	518	25.0	1180	52.9	3313	32.7
Ever drank alcohol during year prior to pregnancy	2382	82.2	N/A	N/A	530	66.5	1948	95.3	1879	84.3	6739	84.6
Ever binge drank during pregnancy^*b*^	304	10.5	N/A	N/A	0	0.0	16	0.8	183	11.7	503	6.9
Race/ethnicity – White	2295	79.4	1672	80.3	673	88.3	1372	66.7	1667	74.9	7679	76.7
Married or living with partner	2745	94.9	2022	96.0	N/A	N/A	1857	97.7	2104	94.5	8728	95.6
Completed high school	2805	97.0	2007	97.3	N/A	N/A	2035	99.9	2133	97.7	8980	97.9
Completed post-secondary	2182	75.5	1810	87.7	N/A	N/A	1980	97.2	1945	89.1	7917	86.3
In paid employment	1705	58.7	N/A	N/A	628	78.8	1873	93.4	1813	81.3	6019	75.8
Income- Below Market Basket Measure^*c*^	237	8.5	185	9.0	84	10.8	183	9.0	225	10.5	914	9.3
Income- Below median household income	1356	48.4	923	44.7	500	64.1	450	22.1	1033	48.4	4262	43.4
Ever smoke cigarettes before pregnancy	N/A	N/A	923	27.3	311	39.0	488	30.3	787	35.4	2156	32.1
Ever smoke cigarettes during pregnancy	337	11.6	52	2.5	52	6.5	101	6.6	284	16.0	826	9.1
Pre-pregnancy BMI > 30	333	11.7	210	11.2	175	22.6	165	8.5	258	12.3	1141	11.9
First pregnancy	1016	35.3	990	47.5	245	30.7	799	39.2	822	36.9	3872	38.6
	**Median**	**Range**	**Median**	**Range**	**Median**	**Range**	**Median**	**Range**	**Median**	**Range**	**Median**	**Range**
Maternal age (years)^*d*^	31	18–47	32	16–45	32	16–47	34	20–51	31	18–47	32	16–51

^a^N = Number of participants from each cohort with non-missing values for the variable ‘ever drank alcohol during pregnancy’

^b^Binge drinking was defined as consuming 5 or more drinks on one occasion in AOF, APrON, FAMILY, and 3D, and as consuming 4 or more drinks on one occasion in OBS

^c^The Market Basket Measure is a measure of low income based on the cost of a specific basket of goods and services representing a modest, basic standard of living developed by Employment and Social Development Canada (ESDC). Reference values for each cohort were taken from the most relevant MBM region available (metropolitan area or province + urbanicity) from the closest census (2015 or 2010) values published by Statistics Canada.* In all cases, percent is calculated out of the non-missing values for the given maternal characteristic, which may differ from the overall N for the cohort

^d^Maternal age is summarized from age at first visit during pregnancy for AOF, FAMILY, OBS, and 3D, and from age at delivery for APrON, Range = Min–Max

### Unadjusted associations with alcohol consumption during pregnancy

The unadjusted associations of the factors included in our model with any alcohol consumption during pregnancy are presented in Table 4, and the same associations for binge drinking during pregnancy in Table 5. These tables also indicate what variables were available, or not available, in each cohort. The largest unadjusted association with alcohol consumption during pregnancy was alcohol consumption prior to pregnancy, with women who consumed alcohol before pregnancy having 32.83 times higher odds of reporting that they consumed some alcohol during pregnancy compared to those women who did not consume alcohol prior to pregnancy (95% CI: 22.91, 47.05). This ratio largely reflects the rarity of women who do not report any alcohol consumption in the year before pregnancy but do report consumption during pregnancy. Smoking during pregnancy, white ethnicity, binge drinking before pregnancy, having a higher income, not being married or living with a partner, and not completing postsecondary education were also associated with alcohol consumption during pregnancy in the unadjusted analysis (Table 5). The largest unadjusted association with any binge drinking during pregnancy was also any alcohol consumption in the year prior to pregnancy, followed by any binge drinking in the year prior to pregnancy (Table 5). Smoking during pregnancy, not completing high school, not being married or living with a partner, not completing post-secondary education, white ethnicity, smoking before pregnancy, and being below the median household income were also positively associated with any binge drinking during pregnancy.

**Table 4:** Unadjusted odds ratios and confidence intervals for any alcohol consumption during pregnancy

	**OR**^**a**^	**95% CI**^**b**^	**AOF**	**APrON**	**FAMILY**	**OBS**	**3D**
Alcohol consumption 1 year before pregnancy	32.83	(22.91, 47.05)	X	-	X	X	X
Smoking during pregnancy	4.02	(3.46, 4.66)	X	X	X	X	X
Race/ethnic background – White	2.63	(2.34, 2.95)	X	X	X	X	X
Binge drinking 1 year before pregnancy^*c*^	2.04	(1.84, 2.26)	X	-	-	X	X
Above Market Basket Measure	1.92	(1.63, 2.27)	X	X	X	X	X
Not married or living with partner	1.83	(1.49, 2.24)	X	X	-	X	X
Smoking before pregnancy	1.78	(1.59, 2.00)	-	X	X	X	X
Above median household income	1.33	(1.22, 1.45)	X	X	X	X	X
Did not complete post-secondary	1.24	(1.10, 1.40)	X	X	-	X	X
Did not complete high school	1.23	(0.92, 1.64)	X	X	-	X	X
Employed	1.04	(0.94, 1.16)	X	-	X	X	X

^a^*OR* Unadjusted odds ratio

^b^*CI* Confidence interval

^c^Binge drinking was defined as consuming 5 or more drinks on one occasion in AOF, APrON, FAMILY, and 3D, and as consuming 4 or more drinks on one occasion in OBS

**Table 5:** Unadjusted odds ratios and confidence intervals for binge drinking during pregnancy

	**OR**^**a**^	**95% CI**^**b**^	**AOF**	**APrON**	**FAMILY**	**OBS**	**3D**
Alcohol consumption 1 year before pregnancy	53.45	(13.31, 214.58)	X	-	X	X	X
Smoking during pregnancy	7.64	(6.21, 9.41)	X	-	X	X	X
Race/ethnic background – White	2.94	(2.21, 3.90)	X	-	X	X	X
Binge drinking 1 year before pregnancy^c^	11.64	(8.36, 16.21)	X	-	-	X	X
Above Market Basket Measure	1.21	(0.87, 1.68)	X	-	X	X	X
Not married or living with partner	3.62	(2.68, 4.89)	X	-	-	X	X
Smoking before pregnancy	2.56	(1.92, 3.42)	-	-	X	X	X
Below median household income	1.28	(1.06, 1.54)	X	-	X	X	X
Did not complete post-secondary	3.00	(2.44, 3.68)	X	-	-	X	X
Did not complete high school	4.34	(2.84, 6.63)	X	-	-	X	X
Employed	1.04	(0.94, 1.16)	X	-	X	X	X

^a^*OR* Unadjusted odds ratio

^b^*CI* Confidence interval

^c^Binge drinking was defined as consuming 5 or more drinks on one occasion in AOF, APrON, FAMILY, and 3D, and as consuming 4 or more drinks on one occasion in OBS

### Multivariable regression and adjusted associations with alcohol consumption during pregnancy

Adjusted associations with alcohol consumption during pregnancy are reported in Table 6. This analysis included fixed effects of alcohol use prior to pregnancy, smoking during pregnancy, ethnicity, income, maternal age, and cohort membership and included 6,570 observations with complete data. Controlling for other variables, the impact of drinking prior to pregnancy remained highly significant and of relatively large effect, with women who consumed alcohol prior to pregnancy having 31.74 times the odds of alcohol consumption during pregnancy compared with those who did not (95% CI: 21.58, 48.96). Similarly, smoking during pregnancy (AOR 2.84; 95% CI: 2.34, 3.46), white ethnicity (AOR 2.04; 95% CI: 1.75, 2.38), and having a higher income (AOR 1.32; 95% CI: 1.02, 1.70) were associated with greater odds of alcohol consumption during pregnancy. Neither the linear nor quadratic term for age was significantly associated with alcohol consumption during pregnancy in the adjusted analysis.

**Table 6:** Adjusted coefficients and odds ratios from multiple regression of factors associated with alcohol consumption during pregnancy

	**AOR**^**a**^	**95% CI**		**coefficient**	**std. error**	**z-value**	***p*-value**
**Cohort**							
OBS	1			1			
3D	4.18	3.52	4.96	1.43	0.09	16.4	< 0.001
AOF	3.94	3.37	4.61	1.37	0.08	17.10	< 0.001
FAMILY	0.16	0.10	0.23	-1.84	0.20	-9.02	< 0.001
**Alcohol use (1 year prior)**							
No	1			1			
Yes	31.74	21.58	48.96	3.46	0.21	16.60	< 0.001
**Smoking (during pregnancy)**							
No	1			1			
Yes	2.84	2.34	3.46	1.04	0.10	10.40	< 0.001
**Race/ Ethnicity**							
Non-white race or ethnicity	1			1			
White	2.04	1.75	2.38	0.71	0.08	9.04	< 0.001
**Income**							
Level 1 – Below MBM	1			1			
Level 2 – Above MBM, below median	1.32	1.02	1.70	0.28	0.13	2.12	0.034
Level 3 – Above median HH income	1.47	1.14	1.89	0.38	0.13	2.96	0.003
**Age**							
Age at pregnancy (years)	1.08	0.95	1.23	0.07	0.07	1.11	0.267
Age﻿^2^				< 0.01	< 0.01	-0.91	0.364

^a^*AOR* Adjusted odds ratio; Model is adjusted for cohort, maternal age, race/ethnicity, alcohol use in the one year prior to pregnancy, smoking during pregnancy, and income

Participants from different cohorts also differed significantly in their risk for alcohol consumption during pregnancy. For example, the adjusted odds ratio of membership in the 3D study compared to membership in OBS was 14.68 (95% CI: 8.48, 28.04), indicating that the odds of a participant in 3D ever reporting alcohol consumption during pregnancy are much higher than a participant in OBS. Examination of interactions between cohort membership and estimated effects suggested that the strength of association between smoking during pregnancy and white ethnicity differed among cohorts (there were significant interactions between cohort membership and risk factors), but the main effects of both remained significant and positive when adjusting for these interactions.

Adjusted associations with any binge drinking during pregnancy are reported in Table 7 (*n* = 6,273) and showed some similar patterns as for alcohol consumption during pregnancy. Alcohol use in the year prior to pregnancy, smoking during pregnancy, and white ethnicity were associated with greater odds of binge drinking during pregnancy, and cohorts differed significantly in risk for binge drinking during pregnancy. Income and age were not predictive of binge drinking during pregnancy. The relatively small number of women reporting binge drinking during pregnancy (*n* = 429) and differences in the prevalence of binge drinking behaviour among cohorts should be noted in interpreting model coefficients; for example, no participants from the FAMILY study reported binge drinking during pregnancy. However, a test for differences among the remaining cohorts in effects of risk factors on binge drinking suggested that there were no significant cohort-by-risk-factor interactions.

**Table 7:** Adjusted coefficients and odds ratios from multiple regression of risk and protective factors for binge drinking during pregnancy

	**AOR**^**a**^	**95% CI**		**coefficient**	**std. error**	**z-value**	***p*-value**
**Cohort**							
OBS	1			1			
3D	14.68	8.48	28.04	2.80	0.311	9.00	< 0.001
AOF	16.50	9.33	31.97	2.69	0.302	8.88	< 0.001
FAMILY^b^	0.00	0.00	0.00	-14.3	364	-0.04	0.969
**Alcohol use (1 year prior)**							
No	1			1			
Yes	40.17	12.76	243.56	3.69	0.71	5.17	< 0.001
**Smoking (during pregnancy)**							
No	1			1			
Yes	5.44	4.28	6.91	1.69	0.12	13.86	< 0.001
**Race/ Ethnicity**							
Non-white race or ethnicity	1			1			
White	1.39	1.01	1.94	0.329	0.16	1.98	0.048
**Income**							
Level 1 – Below MBM	1			1			
Level 2 – Above MBM, below median	1.41	0.91	2.23	0.343	0.23	1.51	0.130
Level 3 – Above median HH income	1.53	0.98	2.45	0.424	0.23	1.81	0.070
**Age**							
Age (in years)	0.82	0.66	1.03	-0.194	0.11	-1.70	0.090
Age^2^				0.002	< 0.01	-0.91	1.25

^a^*AOR* Adjusted odds ratios; Model is adjusted for cohort, maternal age, race/ethnicity, alcohol use in the one year prior to pregnancy, smoking during pregnancy, and income

^b^No participants in FAMILY reported binge drinking during pregnancy

### Impact of alcohol consumption during pregnancy on birth outcomes

Summaries of selected birth outcomes based on Canadian reference values for singleton births across the cohorts are presented in Table 8.

**Table 8:** Frequencies and percentages for preterm and very preterm births and low birthweight percentiles from singleton births across cohorts

	**AOF**		**APrON**		**FAMILY**		**OBS**		**3D**		**Total**	
	(*N* = 3,125)^a^		(*N* = 1,866)		(*N* = 765)		(*N* = 1,347)		(*N* = 2,251)		(*N* = 9,354)	
	**n**	**%**	**n**	**%**	**n**	**%**	**n**	**%**	**n**	**%**	**n**	**%**
Gestational age < 37 weeks	199	6.4	88	4.7	53	6.9	71	5.3	152	6.8	563	6.0
Gestational age < 32 weeks	22	0.7	8	0.4	8	1.1	6	0.5	35	1.6	79	0.8
Birthweight < 10^th^ percentile^b^	375	12.0	232	12.4	68	8.9	179	13.3	264	11.7	1118	12.0
Birthweight < 3^rd^ percentile	96	3.1	59	3.2	19	2.5	35	2.6	75	3.3	284	3.0

^a^ N = Number of participants from each cohort with non-missing values for the harmonized variable ‘gestational age at delivery’

^b^ Percentiles based on reference ranges adjusted for gestational age and infant sex from Canadian singletons described by Kramer et al. 2001 [45]

Table 9 shows the adjusted odds ratios for associations of alcohol use during pregnancy with birth outcomes. Neither any alcohol consumption during pregnancy nor binge drinking during pregnancy were significantly associated with greater adjusted odds for preterm delivery in our sample.

**Table 9:** Adjusted odds ratios and confidence intervals for maternal alcohol use during pregnancy on birth outcomes

Exposure	Outcome	N	AOR^a^	95% CI
**Any alcohol consumption**				
Preterm birth		5,743	1.23	(0.93, 1.63)
Small for gestational age		5,516	1.12	(0.91, 1.37)
**Binge drinking**^**b**^				
Preterm birth		4,554	1.34	(0.84, 2.07)
Small for gestational age		4,329	1.01	(0.71, 1.43)

^a^Model is adjusted for maternal age, race/ethnicity, alcohol use in the one year prior to pregnancy, smoking during pregnancy, income, gravidity, gestational hypertension, and gestational diabetes

^b^Binge drinking was defined as consuming 5 or more drinks on one occasion AOF, APrON, FAMILY and T3D, and 4 or more drinks per occasion for OBS

## Discussion

Using harmonized data across five Canadian pregnancy/birth cohorts, we report on alcohol consumption before and during pregnancy and the impact of alcohol exposure on immediate birth outcomes. Recent research in the field of FASD prevention has emphasized the identification of the prevalence of alcohol use during pregnancy, as well as the factors and influences associated with such use, with results reported from several countries [48–53]. In our harmonized cohort, alcohol consumption during pregnancy was highly associated with [1] alcohol consumption prior to pregnancy and [2] smoking during pregnancy. In adjusted models, alcohol consumption during pregnancy was also associated with higher income, and white ethnicity, although to a lesser magnitude. Neither any alcohol consumption during pregnancy nor binge drinking during pregnancy, as defined in this study, was associated with preterm delivery or low birth weight for gestational age, when adjusted for maternal age, race/ethnicity, alcohol use in the one-year prior to pregnancy, smoking during pregnancy, income, gestational hypertension, and gestational diabetes. While we did not find an association between alcohol use during pregnancy and adverse neonatal outcomes in this study, it is important to emphasize that the possible impacts of PAE extend beyond the neonatal period and indeed persist across the lifespan. Notably, recent mental health research in the field of FASD focusing on suicidality and related biopsychosocial risk factors demonstrated that individuals assessed for FASD did not differ on their experiences of suicidality based on FASD diagnostic outcome (i.e., those who received a diagnosis of FASD did not differ from those who did not receive a formal diagnosis), emphasizing that PAE in and of itself may be a critical factor given the deleterious effects of alcohol on the brain’s stress-response system, coupled with the cumulative psychosocial and environmental adversity often experienced by individuals with FASD [54]. Individuals with FASD have been well documented to have disproportionately high rates of prenatal and postnatal adversity, contributing to high rates of challenges in school, employment, housing, independence, victimization, and legal involvement across the lifespan [55].

Notably, in our sample, the estimated proportion of women who reported any alcohol consumption over the course of pregnancy, including prior to pregnancy recognition, was highly variable, ranging from 3.6% (FAMILY) and 7.2% (APrON) to 42.8% (AOF) and 49.9% (3D). However, the exact magnitude of disparities should be interpreted with caution due to differences in how studies asked participants about alcohol consumption (Supplementary Table S2). Some question formats made it difficult to distinguish between no drinking and rarely drinking, leading to underestimating rates of drinking during pregnancy, while other study formats allowed participants to report even single instances of drinking. Question wordings also differed in consideration of alcohol use prior to pregnancy recognition, with explicit wording to include this likely resulting in higher reported rates than in studies without this wording.

These descriptive findings speak to the variability of how questions related to substance use are asked and implications for information precision and cross-study compatibility, including the ability to obtain information on low-dose alcohol use, differences in women’s behaviour after pregnancy recognition, and women’s alcohol use during pregnancy. These factors are likely influenced by diverse and intersectional social determinants of health. While the cohorts in this study were gathered from three different provinces and include data collected over a relatively large time span (2004–2019), the findings suggest a need to re-visit the prevalence of alcohol use during pregnancy in Canada beyond the consistently reported 10% prevalence rate and to investigate variability in different provinces and demographic groups [25]. Additionally, there is a need to consider the clinical implications of these diverse patterns of alcohol use, both pre-pregnancy and during pregnancy, among further efforts to prevent both current and future alcohol-exposed pregnancies. Given that pre-pregnancy alcohol use is a significant predictor of alcohol use during pregnancy, and that Canadian women of childbearing age report high levels of weekly alcohol consumption (30.5%) and past year heavy drinking (18.3%) [1], understanding the pre-pregnancy alcohol use patterns of women has important implications for screening and brief intervention.

In our study, neither any alcohol consumption during pregnancy nor binge drinking during pregnancy was associated with greater adjusted odds for preterm birth or low birth weight for gestational age, when adjusting for common variables associated with these outcomes including smoking, income and hypertension. This is in contrast to previous research that has identified an increased risk of premature birth [56], stillbirth [57], and low birth weight [58] associated with prenatal alcohol exposure. However, this finding may be explained by the fact that increased risk for adverse birth outcomes has been identified as being dose dependent with heavy alcohol consumption during pregnancy, in contrast to light or moderate alcohol consumption, increasing the risk of these three outcomes [18, 59]. The measure of any alcohol use during pregnancy would include a large proportion of low-dose alcohol use across the pregnancy, and while any binge drinking during pregnancy targets heavier alcohol use, our measure could not differentiate between exposure at different stages of pregnancy or the frequency and magnitude of binge drinking.

The impact of prenatal alcohol exposure on fetal growth is controversial since only a minority of newborns with PAE demonstrate delayed growth and growth restriction is not always present in children with FASD [60, 61]. Recently, growth was removed as a core diagnostic feature for FASD in both Canada [7] and Australia [62]. However, other scholars have argued that growth deficiency is still an essential diagnostic criterion for FASD [63]. It is likely that the impact of PAE on fetal and subsequent newborn growth is dependent upon several factors including, timing, frequency, amount, and duration of exposure which is beyond the scope of this analysis.

Despite the common tendency to see ‘health behaviours’ as a single action, activities such as alcohol consumption are multidimensional areas of human activity [64]. Consuming alcohol during pregnancy is not a singular behaviour; rather, ‘alcohol consumption’ is a cluster of actions with variation in how much is consumed, where, when, and for what reason. Past studies have supported the notion that the practice of alcohol consumption during pregnancy cannot be reduced to one behaviour and those who drink during pregnancy do not represent one group. Older women [20, 65–69] with higher incomes [65, 66, 68–71] and more education [66, 71] are consistently shown to have greater odds of alcohol use during pregnancy, but so are women who have unplanned pregnancies [20, 65, 69], drink heavily before pregnancy [65, 71, 72], and smoke during pregnancy [20, 65, 67, 72, 73], factors more commonly associated with younger age, lower income, and/or less educational attainment [74, 75].

The heterogeneity among the findings across the different cohorts suggests that women who consume alcohol during pregnancy in Canada likely represent populations who differ in lifestyle and consumption patterns and that different populations may have different patterns of alcohol use in terms of their frequency, amount, and timing. The different odds of drinking during pregnancy observed between the cohorts could represent different underlying prevalence rates in the population from which the cohorts were drawn (e.g., province, hospital catchment area) or variation in alcohol use in Canada over time as the data collection periods across the cohorts also varied. An examination of the variability in prevalence rates of alcohol use across cohorts requires further investigation.

### Strengths and limitations

Harmonization across multiple studies provided an opportunity to increase sample sizes and study less frequent outcomes/exposures. Few Canadian studies have been published examining the sociodemographic characteristics associated with alcohol consumption during pregnancy and the impact of alcohol use on adverse birth outcomes, and our study is the largest Canadian sample to date. Pooling data from multiple cohort studies also provided us an opportunity to directly examine the relative sizes of the estimated pooled associations and cohort differences, which revealed the heterogeneity across the cohorts. Fixed effects of cohort membership in the models were consistently large and significant, reflecting the different average tendencies in alcohol use among participants from different cohorts. Cohorts had similar median age (early 30 s), high educational attainment (completed post-secondary range 75.5 to 97.2%), and very high proportion married or living with a partner (range 94.9 to 97.7%). There was variation in household income (below median income range 22.1 to 48.4%) and ethnicity (white range 66.7 to 88.3%) among the cohorts.

However, as our study demonstrated, analyzing pooled data from multiple studies poses many challenges as studies often use different instruments and approaches to measure the same construct. As previously discussed, our analysis was based on the best available data from the five cohorts included. Differences in how and when alcohol use was measured in different cohorts limited the ability to harmonize all variables of interest across cohorts. As a result, we needed to reduce alcohol consumption to a less precise dichotomous measure of any alcohol use during pregnancy versus none. Additionally, we originally aimed to include longitudinal measures of alcohol consumption over the course of pregnancy (e.g., by trimester) and the patterns of alcohol use after pregnancy, but these data were not measured consistently across the cohorts in a way that could be harmonized.

### Directions for future studies

To increase comparability across studies and potential for data reuse, future pregnancy and birth cohorts should include questions related to the frequency and amount of alcohol consumed before and during pregnancy, and not rely only on categorical measures of alcohol use. In cohort studies assessing multiple exposures, there is always a trade-off made as to what information to collect, as it is not feasible to collect detailed information about all variables of interest. However, alcohol consumption should be considered an essential core variable collected at a minimum level of detail. To ensure that questions about PAE can be accurately addressed, questions that assess the frequency of alcohol used using standard categories such as those used in the Alcohol Use Disorders Identification Test (AUDIT) could allow for comparison across cohorts and with other studies that employ the commonly used measure of alcohol use [76]. However, the AUDIT defines binge drinking of “6 or more drinks” and more detail about lower amounts and less frequent drinking are important to collect to assess the outcomes of drinking during pregnancy. The AUDIT may be one helpful tool that could be utilized for collecting consistent information about alcohol use during pregnancy given that it is one of five screening tools developed and/or validated for alcohol use screening among pregnant women [77]. However, all screening and brief intervention practices should be determined based on the individual needs of the individual and the provider, and we emphasize the importance of engaging in conversations with all women about alcohol and substance use [24, 78]. Screening and brief intervention offers important opportunities for, as well as for identifying women who may need further supports and services for their alcohol use.

As we need to better understand the impact of different levels of occasional drinking during pregnancy, the development of standard questions for pregnancy studies is needed. Questions regarding the average quantity of alcohol consumed on each occasion should be a continuous number that can be re-categorized as necessary. Additionally, asking about alcohol use prior to pregnancy using the same response categories of frequency and quantity and about the timing of pregnancy recognition would allow the determination of changes in use following pregnancy recognition. When linked to birth outcomes, this information can provide critical information to understanding risk, for example, the risk associated with heavy preconception use, as well as important information about critical and sensitive periods of PAE [17]. This information would also provide enhanced clarity in further investigations into neonatal outcomes, given the often-contradictory nature of the evidence, particularly regarding low birthweight and preterm birth.

Finally, person-centred analysis techniques such as cluster analysis, latent profile analysis, and latent class modelling can be used to identify important subgroups within a larger population [79, 80]. The field of alcohol-exposed pregnancies has recently benefited from the use of such methods to identify different patterns and trajectories of alcohol use within a larger population of women who drink during pregnancy [68, 81, 82]. However, to conduct such an analysis, information about the amount and timing of alcohol exposure is necessary.

## Conclusions

Our study harmonized data from five Canadian pregnancy cohorts and reports on alcohol use before and during pregnancy from the resulting large sample of pregnant women. Women’s alcohol use during pregnancy was highly associated with alcohol consumption prior to pregnancy and smoking during pregnancy. It was also associated with higher income and white ethnicity. Neither any drinking during pregnancy nor binge drinking during pregnancy was associated with preterm delivery or low birth weight for gestational age in our sample. Due to the significant diversity in the way in which birth cohorts solicited information about alcohol use, we were only able to include alcohol use as a dichotomous (yes/no) behaviour. A conclusive clinical message is therefore difficult to make based on the existing Canadian data. Greater consistency in the way information about frequency, timing and amount of alcohol consumed before pregnancy, prior to pregnancy recognition and during pregnancy is gathered would be required in future pregnancy and birth cohorts to enhance the compatibility and re-usability of collected data for collaborative analyses.

## Supplementary Information

**Additional file 1:** **Supplementary Table S1.** Harmonized variables created for the current study and possibility to generate each variable across participating cohorts. **Supplementary Table S2. **Format of questions about alcohol consumption during pregnancy in different cohorts.

## Data Availability

Documentation of the harmonization project, the participating studies and data that they collected, and the harmonized dataset produced in this study are available from the Maelstrom Research catalogue (https://www.maelstrom-research.org/study/reach-pae-hi), and further information about the ReACH network is available from the ReACH catalogue (https://www.maelstrom-research.org/network/reach). Use of individual participant data in the current study was requested from and approved by individual cohort studies as well as relevant ethics committees of the authors’ institutions. Individual participant data used in this study are not publicly available. Access to data collected by studies must be requested through the principal investigator or access committee of each study (AOF: contact Muci Wu (University of Calgary, muci.wu@ucalgary.ca) or visit http://allourfamiliesstudy.com/; APrON: contact Andrea Deane (Cumming School of Medicine, University of Calgary, Andrea.Deane@albertahealthservices.ca) or visit https://apronstudy.ca/contact-us/; FAMILY: contact Professor Koon Teo (PHRI, McMaster University, koon.teo@phri.ca); OBS: visit http://www.ontariobirthstudy.ca/; 3D: visit https://www.irnpqeo.ca/en/; or click on links under “Participating Studies” at https://www.maelstrom-research.org/study/reach-pae-hi to see documentation about “Access” for each study).

## Abbreviations

PAE: Prenatal Alcohol Exposure
FASD: Fetal Alcohol Spectrum Disorder

## Footnotes

1 People of other genders can experience pregnancy; however, most literature about PAE has either included only women, or has not collected/reported the gender of participants. As such, we will use the term “women” in this paper.
