# Supplementary material for: A harmonized analysis of five Canadian pregnancy cohort studies: exploring the characteristics and pregnancy outcomes associated with prenatal alcohol exposure
Source: BMC Pregnancy Childbirth. 2023 Feb 28;23:128. doi: 10.1186/s12884-023-05447-2 (PMC9972615; doi:10.1186/s12884-023-05447-2)
Supplement: Supplementary file 1 — Additional file 1: Supplementary Table S1. Harmonized variables created for the current study and possibility to generate each variable across participating cohorts. Supplementary Table S2. Format of questions about alcohol consumption during pregnancy in different cohorts. [file 12884_2023_5447_MOESM1_ESM.docx]

***Supplementary Table S1: Harmonized variables created for the current study and possibility to generate each variable across participating cohorts***

|  |  |  |  | **Cohort** | | | | |  |
| --- | --- | --- | --- | --- | --- | --- | --- | --- | --- |
| **Label** | **Domain of information** | **Type of value** | **Categories** | **AOF** | **APrON** | **FAMILY** | **OBS** | **3D** | |
| Alcohol consumption by mother before pregnancy | Lifestyle & behaviours | Integer | 0: Did not consume alcohol  1: Did consume alcohol | 0 | 1 | 0 | 1 | 0 | |
| Alcohol consumption by mother 1 year prior to pregnancy | Lifestyle & behaviours | Integer | 0: Did not consume alcohol  1: Did consume alcohol | 1 | 0 | 1 | 1 | 1 | |
| Alcohol consumption by mother during pregnancy^a^ | Lifestyle & behaviours | Integer | 0: Did not consume alcohol 1: Did consume alcohol | 1 | 1 | 1 | 1 | 1 | |
| Binge drinking by mother 1 year prior to pregnancy | Lifestyle & behaviours | Integer | 0: Did not binge drink 1: Did binge drink | 1 | 0 | 0 | 1 | 1 | |
| Binge drinking by mother during pregnancy | Lifestyle & behaviours | Integer | 0: Did not binge drink 1: Did binge drink | 1 | 0 | 1 | 1 | 1 | |
| Definition of binge drinking | Administrative information | Integer | 1: 4 or more drinks 2: 5 or more drinks | 1 | 0 | 1 | 1 | 1 | |
| Cigarette smoking by mother before pregnancy | Lifestyle & behaviours | Integer | 0: Did not smoke cigarettes 1: Did smoke cigarettes | 0 | 1 | 1 | 1 | 1 | |
| Cigarettes smoking by mother 1 year prior to pregnancy | Lifestyle & behaviours | Integer | 0: Did not smoke cigarettes 1: Did smoke cigarettes | 1 | 0 | 0 | 0 | 1 | |
| Cigarette smoking by mother during pregnancy | Lifestyle & behaviours | Integer | 0: Does not smoke cigarettes 1: Does smoke cigarettes | 1 | 1 | 1 | 1 | 1 | |
| Cigarette smoking cessation by mother during pregnancy | Lifestyle & behaviours | Integer | 0: Did stop smoking cigarettes 1: Did not stop smoking cigarettes | 0 | 1 | 0 | 1 | 1 | |
| Mother's age at 1st pregnancy visit | Reproduction | Integer |  | 1 | 1 | 1 | 1 | 1 | |
| Mother's age at delivery | Reproduction | Integer |  | 1 | 0 | 1 | 0 | 1 | |
| Mother's date of birth | Sociodemographic & economic  characteristics | Date |  | 0 | 0 | 1 | 0 | 1 | |
| Baby 1: Sex | Sociodemographic & economic  characteristics | Integer | 1: Male 2: Female | 1 | 1 | 1 | 1 | 1 | |
| Baby 2: Sex | Sociodemographic & economic  characteristics | Integer | 1: Male 2: Female | 1 | 1 | 1 | 1 | 0 | |
| Baby 3: Sex | Sociodemographic & economic  characteristics | Integer | 1: Male 2: Female | 0 | 0 | 1 | 0 | 0 | |
| Mother's ethnic background - White | Sociodemographic & economic  characteristics | Integer | 0: Not White (European descent) 1: White (European descent) | 1 | 1 | 1 | 1 | 1 | |
| Mother's ethnic background - Aboriginal^b^ | Sociodemographic & economic  characteristics | Integer | 0: Not Aboriginal (e.g., First Nations, Métis, Inuit) 1: Aboriginal (e.g., First Nations, Métis, Inuit) | 1 | 1 | 1 | 1 | 1 | |
| Mother's ethnic background - Black | Sociodemographic & economic  characteristics | Integer | 0: Not Black (African or Caribbean descent)  1: Black (African or Caribbean descent) | 1 | 1 | 1 | 1 | 1 | |
| Mother's ethnic background - South Asian | Sociodemographic & economic  characteristics | Integer | 0: Not South Asian (e.g., India, Sri Lanka, Pakistan, Bangladesh) 1: South Asian (e.g., India, Sri Lanka, Pakistan, Bangladesh) | 1 | 1 | 1 | 1 | 1 | |
| Mother's ethnic background - Latin | Sociodemographic & economic characteristics | Integer | 0: Not Latin American/Hispanic 1: Latin American/Hispanic | 1 | 1 | 1 | 1 | 1 | |
| Mother's ethnic background - Other | Sociodemographic & economic characteristics | Integer | 0: Not Other ethnic group 1: Other ethnic group | 1 | 1 | 1 | 1 | 1 | |
| Civil status at time of questionnaire | Sociodemographic & economic characteristics | Integer | 0: Single or never married  1: Married or living with partner 2: Divorced or separated 3: Widowed | 1 | 1 | 0 | 1 | 1 | |
| Mother's employment status at time of questionnaire | Sociodemographic & economic characteristics | Integer | 0: Not in paid employment  1: In paid employment | 1 | 0 | 1 | 1 | 1 | |
| Average hours mother works in a week at time of questionnaire | Sociodemographic & economic characteristics | Integer |  | 1 | 0 | 1 | 0 | 1 | |
| Market Basket Measure status^c^ | Sociodemographic & economic characteristics | Integer | 0: Above MBM 1: Below MBM | 1 | 1 | 1 | 1 | 1 | |
| Below median household income | Sociodemographic & economic characteristics | Integer | 0: Above median household income 1: Below median household income | 1 | 1 | 1 | 1 | 1 | |
| Completed elementary school | Sociodemographic & economic characteristics | Integer | 0: Did not complete at least elementary school 1: Completed at least elementary school | 0 | 0 | 0 | 1 | 1 | |
| Completed high school | Sociodemographic & economic characteristics | Integer | 0: Did not complete high school 1: Completed at least high school | 1 | 1 | 0 | 1 | 1 | |
| Completed below Bachelor's degree | Sociodemographic & economic characteristics | Integer | 0: Did not complete any post-secondary below Bachelor's degree 1: Completed at least some post-secondary below Bachelor's degree | 0 | 1 | 0 | 1 | 1 | |
| Completed college/trade/university | Sociodemographic & economic characteristics | Integer | 0: Did not complete some post-secondary education 1: Did complete some post-secondary education | 1 | 1 | 0 | 1 | 1 | |
| Completed university | Sociodemographic & economic characteristics | Integer | 0: Did not complete Bachelor's degree 1: Completed at least Bachelor's degree | 0 | 1 | 0 | 1 | 1 | |
| Completed graduate school | Sociodemographic & economic characteristics | Integer | 0: Did not complete graduate school 1: Completed graduate school | 1 | 1 | 0 | 1 | 1 | |
| BMI of mother before pregnancy | Physical measures | Decimal |  | 1 | 1 | 1 | 1 | 1 | |
| BMI of mother before pregnancy > 30 | Physical measures | Integer | 0: Pre-pregnancy maternal BMI not greater than 30 1: Pre-pregnancy maternal BMI greater than 30 | 1 | 1 | 1 | 1 | 1 | |
| Height of mother | Physical measures | Decimal |  | 1 | 1 | 1 | 1 | 1 | |
| Weight of mother before pregnancy | Physical measures | Decimal |  | 1 | 1 | 1 | 1 | 1 | |
| Baby 1: Birth weight | Physical measures | Decimal |  | 1 | 1 | 1 | 1 | 1 | |
| Baby 2: Birth weight | Physical measures | Decimal |  | 1 | 1 | 1 | 1 | 0 | |
| Baby 3: Birth weight | Physical measures | Decimal |  | 0 | 1 | 1 | 0 | 0 | |
| Multiple birth | Reproduction | Integer | 0: Not multiple birth 1: Multiple birth | 1 | 1 | 1 | 1 | 1 | |
| Baby 1: Outcome of the pregnancy | Reproduction | Integer | 0: Live birth 1: Not a live birth | 1 | 1 | 1 | 1 | 1 | |
| Baby 2: Outcome of the pregnancy | Reproduction | Integer | 0: Live birth 1: Not a live birth | 0 | 1 | 1 | 1 | 0 | |
| Baby 3: Outcome of the pregnancy | Reproduction | Integer | 0: Live birth 1: Not a live birth | 0 | 1 | 1 | 0 | 0 | |
| Gestational Age | Reproduction | Integer |  | 1 | 1 | 1 | 1 | 1 | |
| Delivery date | Reproduction | Date |  | 0 | 0 | 1 | 1 | 1 | |
| Preterm birth | Reproduction | Integer | 0: Gestational age at delivery not less than 37 weeks 1: Gestational age at delivery less than 37 weeks | 1 | 1 | 1 | 1 | 1 | |
| Low birth weight for gestational age | Reproduction | Integer | 0: Not low birth weight for gestational age and sex 1: Low birth weight for gestational age and sex | 1 | 1 | 1 | 1 | 1 | |
| Gestational high blood pressure or hypertension | Diseases | Textual | 0: No gestational high blood pressure or hypertension 1: Gestational high blood pressure or hypertension | 1 | 1 | 1 | 1 | 1 | |
| Gestational diabetes | Diseases | Textual | 0: No gestational diabetes 1: Gestational diabetes | 1 | 1 | 1 | 1 | 1 | |
| Pre-eclampsia or eclampsia | Diseases | Textual | 0: No Pre-eclampsia or eclampsia 1: Pre-eclampsia or eclampsia | 1 | 1 | 1 | 1 | 0 | |
| Gravidity | Reproduction | Integer |  | 1 | 1 | 1 | 1 | 1 | |
| Total live births | Reproduction | Integer |  | 1 | 1 | 0 | 1 | 1 | |
| Participant identifier | Administrative information | Textual |  | 1 | 1 | 1 | 1 | 1 | |
| Survey ID | Administrative information | Textual |  | 1 | 1 | 1 | 1 | 1 | |
| Data collection events during pregnancy | Administrative information | Integer | 1: One visit 2: Two visits 3: Three visits | 1 | 1 | 1 | 1 | 1 | |
| Visit 1 attended by mother | Administrative information | Integer | 0: Did not attend first visit 1: Did attend first visit | 1 | 1 | 1 | 1 | 1 | |
| Visit 2 attended by mother | Administrative information | Integer | 0: Did not attend second visit 1: Did attend second visit | 1 | 1 | 0 | 1 | 1 | |
| Visit 3 attended by mother | Administrative information | Integer | 0: Did not attend third visit 1: Did attend third visit | 1 | 1 | 0 | 0 | 1 | |

*a. Due to questionnaire wording, this variable could include alcohol consumption prior to pregnancy recognition, either explicitly (e.g., AOF: “Since becoming pregnant (including before you knew you were pregnant), did you drink any alcohol?”) or implicitly (e.g., 3D: “Since you have become pregnant, how often did you drink alcoholic beverages?”; FAMILY: “During this pregnancy, how often do you drink alcohol in a month?”; OBS: “Currently, during your pregnancy , how many drinks did you have on average during a typical week?”).*

*b. While the term “Indigenous” is more commonly used in the Canadian literature, the label “Aboriginal” is used here as it was originally used in the definition of the DataSchema variable.*

*c. The Market Basket Measure defined by Statistics Canada: “Market Basket Measure refers to the measure of low income based on the cost of a specific basket of goods and services representing a modest, basic standard of living developed by Employment and Social Development Canada (ESDC). The threshold represents the costs of specified qualities and quantities of food, clothing, footwear, transportation, shelter and other expenses for a reference family of two adults and two children. The square root of economic family size is the equivalence scale used to adjust the MBM thresholds for other family sizes.” More details are available on the Statistics Canada website: https://www12.statcan.gc.ca/census-recensement/2016/ref/dict/pop165-eng.cfm.*

***Supplementary Table S2: Format of questions about alcohol consumption during pregnancy in different cohorts.***

| **Cohort** | **Data Collection Event(s)** | **Questions** |
| --- | --- | --- |
| **AOF** | Visit 2:  36 weeks | Since becoming pregnant (including before you knew you were pregnant), did you drink any alcohol?   - Yes - No |
|  |  | Before you knew you were pregnant, how many days per week did you drink alcohol (on average)?   - Less than 1 - 1 - 2 - 3 - 4 - 5 - 6 - 7 |
|  |  | How many drinks would you typically have when you drank (on average)?   - Less than 1 - 2 - 3 - 4 - 5 or more |
|  |  | Once you knew you were pregnant, how many days per week did you drink alcohol (on average)?   - Less than 1 - 1 - 2 - 3 - 4 - 5 - 6 - 7 |
|  |  | How many drinks would you typically have when you drank (on average)?   - Less than 1 - 2 - 3 - 4 - 5 or more |
|  |  | Since becoming pregnant (including before you knew you were pregnant), did you ever drink 5 or more drinks on any one occasion?   - Yes - No |
| **APrON** | Visit 1:  1^st^ trimester  Visit 2:  2^nd^ trimester  Visit 3:  3^rd^ trimester | [Select] I currently drink, on average: |
|  |  | _ _ # drinks per [select] __ day __ week __ month __ year |
|  |  | Comments: ____ |
| **FAMILY** | Visit 1:  21-39 weeks | During this pregnancy, how often do you drink alcohol in a month?   - Never, < 1 a month - Once a month - 2-3 times a month - Once a week - 2-3 times a week - 4-6 times a week - Everyday |
|  |  | During this pregnancy, have you consumed at least 5 or more drinks of alcohol in a day?   - No - Yes |
|  |  | During this pregnancy, how many time have you consumed at least 5 or more drinks of alcohol in a day? |
| **OBS** | Visit 1:  12-16 weeks | Currently, during your pregnancy, how many drinks of [Red wine, White wine, Beer, Liquor/Spirits, Other alcohol] did you have on average during a typical week?   - One or more per week - Less than 1 per week - None - Don't know - Prefer not to answer |
|  |  | *[If One or more per week]* Please specify the number [of red wine, white wine, beer, liquor/spirits, other alcohol] drinks per week you had on a typical week currently during your pregnancy. |
|  |  | Currently, during your pregnancy, how often did you have four or more drinks at the same sitting or occasion?   - One or more times per month - Less than once per month - None - Don't know - Prefer not to answer |
|  |  | *[If One or more times per month]* Please specify the number of times per month you have four or more drinks at the same sitting or occasion (during this pregnancy). |
|  | Visit 2:   28-32 weeks | Have you consumed alcohol over the past 3 months?   - Yes - No - Prefer not to answer |
|  |  | Over the past 3 months, how often did you drink alcohol?   - 6 to 7 times a week - 4 to 5 times a week - 2 to 3 times a week - Once a week - 2 to 3 times a month - About once a month - Less than monthly - Prefer not to answer |
|  |  | Over the past 3 months, on average, how many drinks did you have during a typical week?  Kind of drinks during a typical week: [Red Wine; White Wine; Beer; Liquor/Spirits; Other Alcohol]   - [select to enter the number of drinks per week] - None - Don't know - Prefer not to answer   Drinks per week of [Red wine; White wine; Beer; Liquor/Spirits; Other alcohol] |
| **3D** | Visit 1:  8-14 weeks  Visit 2: 20-24 weeks  Visit 3: 32-35 weeks | Thinking back over the past week, that is, from [date last week] to yesterday, did you have a drink of beer, wine, liquor or any other alcoholic beverage?   - No - Yes - Refuse to answer - Don't know |
|  |  | Starting with yesterday, that is [day name], how many drinks did you have?  Sunday _ _  Monday _ _  Tuesday _ _  Wednesday _ _  Thursday _ _  Friday _ _  Saturday _ _   - Refuse to answer - Don't know |
|  |  | Was there any special occasion during this last week?   - No - Yes |
|  |  | *[Visit 1]* Since you have become pregnant, i.e., since the first day of your last menstruations, was your alcohol consumption more, about the same, or less, compared to your usual alcohol consumption? /  *[Visits 2, 3]* Was your alcohol consumption over the last week more, about the same, or less, compared to the consumption you usually had before your last visit?   - More - About the same - Less - Refuse to answer - Don't know |
|  |  | *[Visit 1]* Since you have become pregnant, / *[Visits 2, 3]* Since your last visit, how often did you…  Drink alcoholic beverages?   - Days of the week **or** - Days of the month **or** - Total number of days since *[Visit 1]* the beginning of your pregnancy / *[Visits 2, 3]* your last visit - Don’t know - Refuse to answer   Have 5 or more drinks on one occasion?   - Days of the week **or** - Days of the month **or** - Total number of days since *[Visit 1]* the beginning of your pregnancy / *[Visits 2, 3]* your last visit - Don’t know - Refuse to answer |
|  |  | On the days that you drank, *[Visit 1]* since you have become pregnant, / *[Visits 2, 3]* since your last visit, how many drinks did you usually have?  _ _ Drink(s)   - Don’t know - Refuse to answer |
|  |  | I understand that you generally do not drink since you are pregnant, but have you had an occasional drink on special occasions like birthday celebrations or family reunions?   - No - Yes - Don’t know - Refuse to answer |
|  |  | How many drinks did you have on those special occasions?  _ _ Drink(s)   - Don’t know - Refuse to answer |
|  |  | How many times did it happen?  _ _ Occasion(s)   - Don’t know - Refuse to answer |
